# Comparing bowel lengthening procedures: which, when, and why?

**DOI:** 10.1097/MOT.0000000000000957

**Published:** 2022-02-09

**Authors:** Jasper B. van Praagh, H. Sijbrand Hofker, Jan-Willem Haveman

**Affiliations:** Department of Surgery, University Medical Center Groningen, University of Groningen, Groningen, The Netherlands

**Keywords:** Intestinal failure, intestinal lengthening, short bowel syndrome

## Abstract

Intestinal failure secondary to short bowel syndrome is still a very serious condition. Treatment consists of parenteral nutrition to provide nutrients and maintain body weight. During the last decades, intestinal lengthening procedures have become more available. The goal of this review is to discuss the results of the literature on the most commonly performed intestinal lengthening procedures.

**Recent findings:**

Longitudinal Intestinal Lengthening, Serial Transverse Enteroplasty (STEP), and Spiral Intestinal Lengthening and Tailoring (SILT) are currently the most frequently reported intestinal lengthening procedures. The most recent literature of these procedures is described with respect to indication, technical details, complications, short and long-term outcome, and PN independence.

**Summary:**

On the basis of indication, surgical complexity, complications, and clinical success, we conclude that the STEP procedure is probably the best choice for most centers.

## INTRODUCTION

Short bowel syndrome is a serious condition with considerable morbidity and mortality. Parenteral nutrition (PN) is the standard treatment in these patients in order to provide nutrients and maintain body weight. Despite the improvement in the care of patients that are chronically PN dependent, it still has several major complications. The most important being central line infections, central vein thrombosis, and chronic liver failure. Furthermore, patients on PN have a low quality of life, comparable with chronic kidney dialysis patients. Therefore, the main goal of (surgical) therapy in these patients should always be to obtain enteral autonomy. 

**Box 1:**
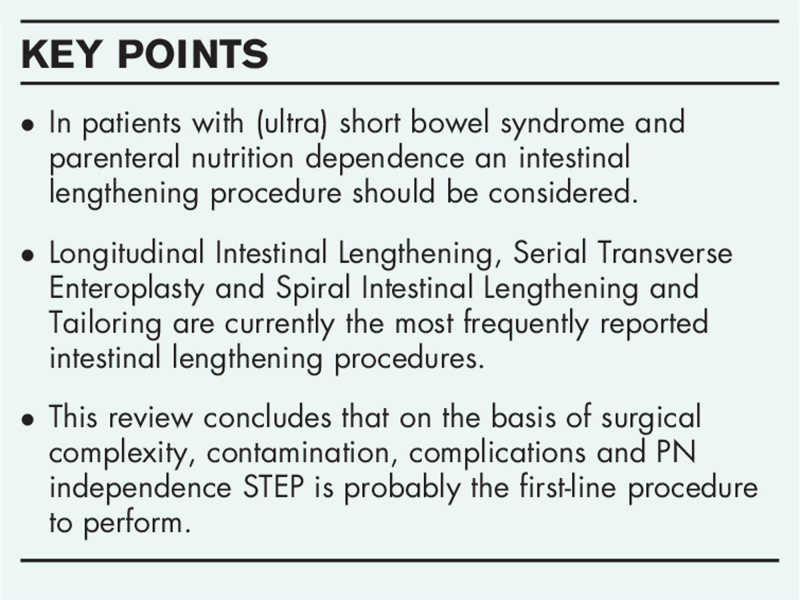
no caption available

A short bowel commonly dilates in diameter which in turn predisposes the remaining bowel for bacterial overgrowth. This results in even less absorption of nutrients. Fortunately, in turn, this dilation can be used for lengthening procedures that may increase the nutritional absorption and even help the patient reach enteral autonomy. Nowadays there are three widely used intestinal lengthening procedures; Longitudinal Intestinal Lengthening and Tailoring (LILT) or Bianchi, Serial Transverse Enteroplasty (STEP), and Spiral Intestinal Lengthening and Tailoring (SILT). Other procedures containing antiperistaltic segments, colon interposition, and nipple valve reconstruction will not be discussed in this review.

The goal of this review is to describe the most recent data on intestinal lengthening procedures and to compare these procedures with respect to complexity, complications, and outcome on PN independence.

## LONGITUDINAL INTESTINAL LENGTHENING AND TAILORING (LILT)

### Short introduction and technique

**FIGURE 1:**
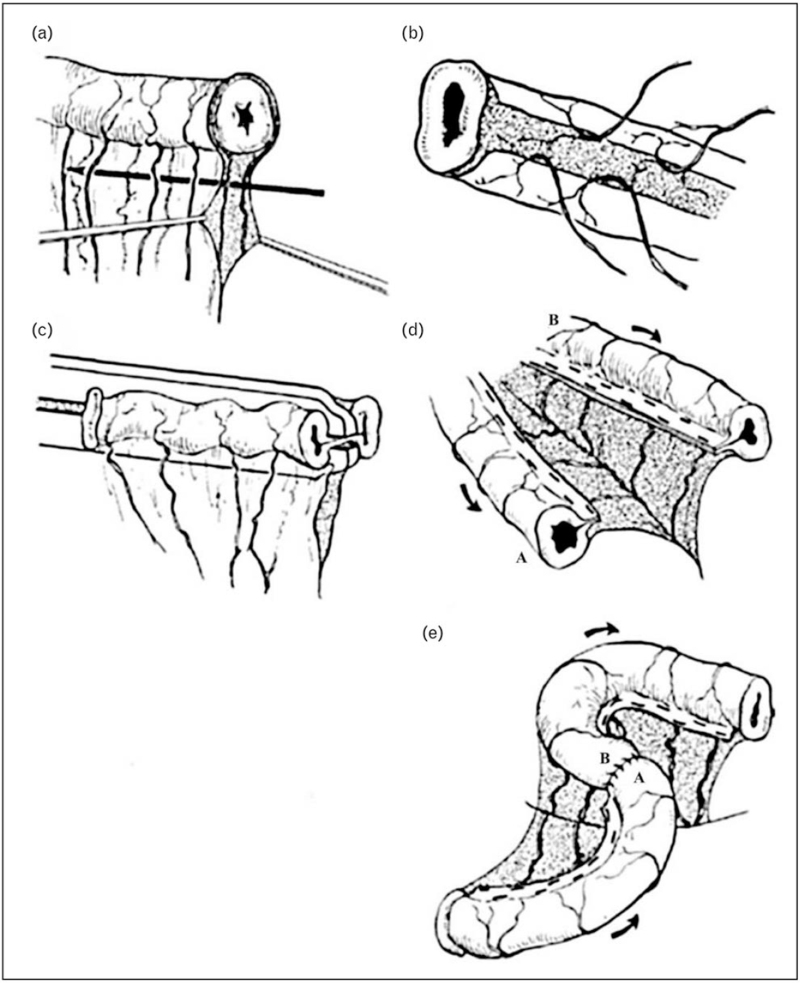
Bianchi procedure or LILT. (a) separating the two leaves of the mesentery of the isolated small bowel segment. (b) Creating a funnel on the mesenteric site for dividing the small bowel. (c), (d) Separating the small bowel by introducing a surgical stapler. This can also be done by cutting the bowel half. (e) The two bowel loops are then anastomosed together in an isoperistaltic manner. ILT, Longitudinal Intestinal Lengthening. Reproduced with permission [1].

In 1980 Bianchi published his first paper on the LILT procedure [1]. In short, the procedure starts with the blunt division of the peritoneal leaves of the small bowel mesentery, then the dilated small intestine is divided longitudinally along the mesenteric and antimesenteric walls. Each of these halves is then tubulized into two parallel pieces that are joined together in an end-to-end isoperistaltic fashion (Fig. 1). In the years after Bianchi's research in pigs [1], several other papers were published with promising results in patients [2].

This technique is quite difficult and needs an intestinal diameter of >3 cm. The residual bowel length needs to be more than 40 cm and a length of dilated bowel loops >20 cm, because of a significant increase in complications in shorter segments. The LILT procedure, in theory, doubles the length of the remaining bowel and preserves bowel vascularization. Another advantage of LILT is that it reduces bacterial overgrowth, but this positive effect is not unique for LILT as it is also described after STEP. Finally, it keeps options open for subsequent other lengthening procedures.

### Outcome and complications

There's not much recent literature that describes the outcomes or complications of LILT. The latest review on LILT sums up research showing that a patient has a chance of 55,5–100% to become PN independent within 2 years after undergoing a LILT procedure [3^▪^]. A recent single-center retrospective study by Shah *et al.* shows similar results (5 out of 9 patients weaned off). In this study, an average bowel length of 30 cm was gained, and it took 9 days before initial feeding was reached [4].

The most feared complication is necrosis of one or both of the created bowel limbs, which can lead to less functional bowel and ultimately to a worse situation than beforehand. Fortunately, no cases with this adverse event have been reported in the recent literature. Other frequently occurring complications are, amongst others, small bowel obstruction (secondary to adhesions), anastomotic strictures, leakages, and fistulas. All these complications can potentially lead to sepsis, liver failure, and mortality [5]. Adequate mortality rates haven’t been reported. This may be caused by the fact that mortality in patients undergoing any lengthening procedure is often multifactorial and mostly not due to surgery (alone).

### Perspective

This technique was ground-breaking at the time that Bianchi described it. Since the first papers in the years following the introduction of LILT, the production of literature (and probably the use of this procedure) has decreased over time. The most recent paper on LILT that we could find is a single-center retrospective study performed by Shah *et al.* where also the STEP procedure is evaluated [4]. The lack of recent literature is not surprising, as it is technically very challenging and, as described below, has few advantages over the STEP procedure.

This type of surgery is not applicable when the mesenteric vessels are compromised and can’t be performed after another lengthening procedure, therefore may be performed as the primary lengthening procedure. STEP after LILT is an option.

## SERIAL TRANSVERSE ENTEROPLASTY

### Short introduction and technique

**FIGURE 2:**
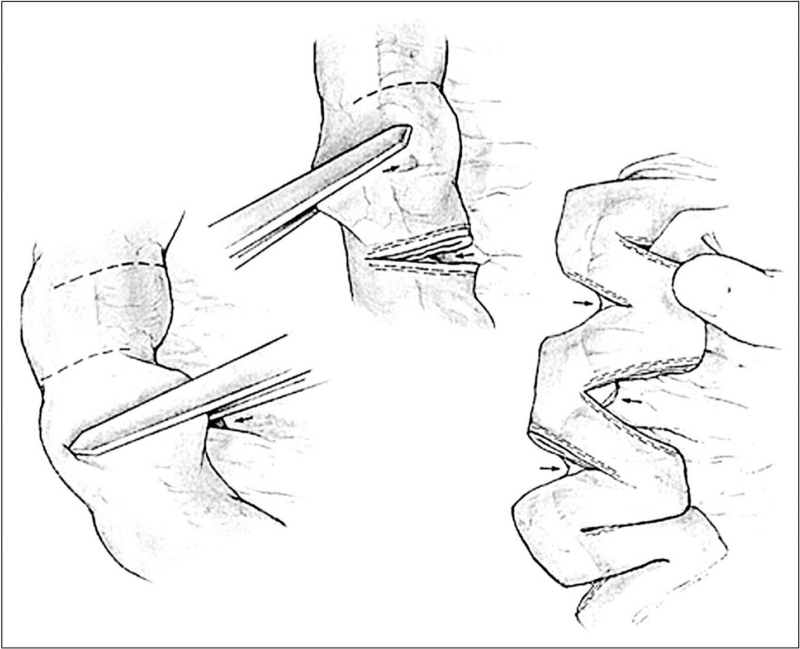
Schematic view of STEP. Perpendicular to the longitudinal axis, a stapler line is made preserving a 2 cm luminal diameter. After multiple staples, the bowel is lengthened. STEP, Serial Transverse Enteroplasty. Reproduced with permission [27].

The STEP procedure is a technique that can be used in any length of the bowel, although the bowel needs to be dilated. In a STEP procedure, a linear stapler is used and then a zig-zag pattern is created with staples opposite to each other. In order to maintain a luminal diameter of 2 cm, the distance between each stapler is approximately 2–2.5 cm. There is no minimal length of bowel necessary, but a minimal lumen diameter of 3.5–4 cm is needed (Fig. 2). Another advantage is that STEP can be used on the duodenum as well. Manipulation to the mesentery is minimal, the bowel is not opened during the procedure, so contamination is limited. Also, STEP is considered technically less challenging than LILT and can be repeated on the same bowel again. Although it is questioned whether this results in better outcomes [6].

### Outcome and complications

An elongation of on average 75% is achieved according to a recent analysis of 19 studies [3^▪^]. A comparative retrospective study showed an increase in length of 25 cm and initial feeding time of 8 days, however without differences in outcome compared to LILT [4]. The chances for patients to be weaned off PN ranges from 6–67% two years after surgery, with an average of 43% [3^▪^]. Another systematic review shows 10 studies (with a minimum follow-up period of 2 years) in which 42% of the patients were weaned from PN [7]. A retrospective study over two eras (2003–2005 and 2006–2016), in which patients with shorter lengths of the bowel before STEP were included in the second era, showed an interesting comparison over time. Overall, in this paper, 42% of patients reached enteral autonomy. It also reported less complications of the procedure in the latter era [8]. A Turkish study showed that 64% had successfully progressed to enteral autonomy vs 27% in their control group without surgery [9]. Complications that are described are staple line leaks, bleeding, strictures or obstructions and abdominal abscess formation [8].

### Perspective

This procedure is probably the most performed lengthening procedure worldwide and recent literature is less scarce, however, there are still few papers that describe recent experience with STEP. As mentioned, STEP can be performed after other intestinal lengthening procedures and has even been done multiple times on the same patients, however with varying success rates [6,10,11]. An interesting study shows a simulation model that concludes that STEP was associated with an increased rate of enteral autonomy compared to transplantation (no STEP beforehand) and that transplantational rates were reduced by STEP [12]. It also has a long-lasting influence on bacterial overgrowth and D-lactic acidosis [13].

## SPIRAL INTESTINAL LENGTHENING AND TAILORING

### Short introduction and technique

**FIGURE 3:**
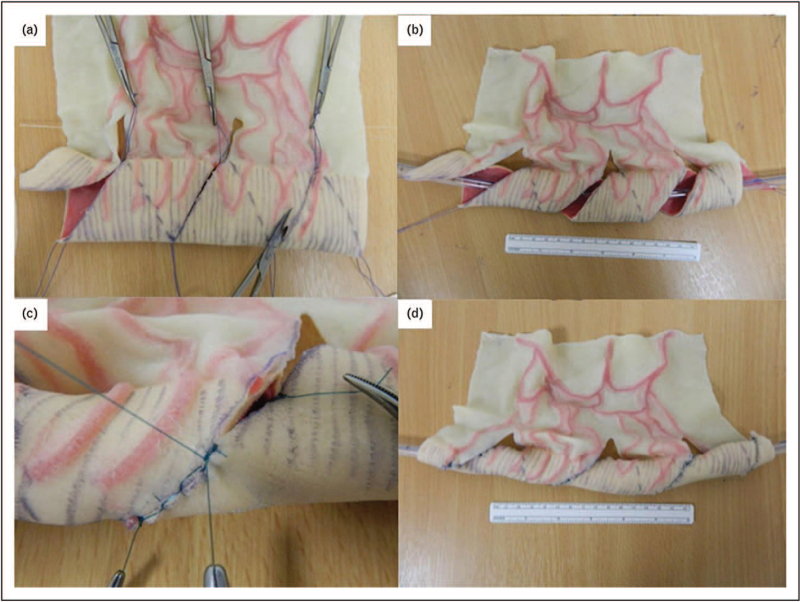
A model of the SILT technique. (a) The bowel is cut spirally in an angle to the longitudinal axis. (b) maintaining orientation by using a silicon catheter, the bowel is stretched. (c) When adjusted to the right length and diameter, the lumen is closed by suturing. (d) The bowel in a longer and narrower shape. SILT, Spiral Intestinal Lengthening and Tailoring. Reproduced with permission [28].

SILT is a relatively new technique that was introduced by Cersni in 2011 [14]. In SILT the mesentery is carefully incised without damaging the vessels, next the intestine is spirally cut. Then the bowel is stretched and closed again over a tube increasing its length and adjusting the luminal diameter (Fig. 3). The main advantage of SILT is that it can be performed when there is a lesser degree of bowel dilatation (≤ 4 cm). Furthermore, the muscle fiber orientation is not altered, unlike STEP were this is altered, which makes the bowel more prone to recurrent dilatation. The authors describe less mesenteric handling than LILT and also less than STEP, although it is mandatory to open the bowel, so there is significant contamination.

### Outcome and complications

In the last two years, there was only one paper to be found that describe their results on SILT [15]. In this series, 5 children are described that were operated on in 5 years time. The median increase in small bowel length was 56%, with tailoring the segment with a 50% decrease in diameter. In two patients SILT was combined with duodenal antimesenteric stapling to tailor the dilated duodenum and in another patients SILT of a less dilated distal jejunum was combined with STEP of the proximal jejunum. No major complications like leakage, necrosis, fistula, obstruction or intra-abdominal abscess were described. Median PN requirement decreased from a median of 7 nights per week to 4 nights. Liver function was preserved or improved, and weight increased. One other paper mentions the use of SILT but lacks an adequate analysis of the results of this procedure [16^▪^].

In addition to this clinical study, Coletta also described a mathematical model that can be used by surgeons in the preoperative planning to calculate the bowel length after reconstruction on the basis of several clinical and surgical parameters [17].

### Perspective

SILT is a valuable alternative to SILT and STEP. Its main advantage is that it can be used in nondilated bowel as well. Furthermore, the orientation of the muscle fibers remains in the anatomical direction. Worldwide experience with SILT in a clinical setting is still scarce, over the last years only very occasionally groups describe their experience in SILT. The lack of experience is also illustrated by the results of a survey that was send to all members of the European Pediatric Surgeon’ Association, in this survey no surgeon mentioned to offer SILT to their patients, moreover, SILT or spiral is not even mentioned in the paper [18^▪^]. Considering the apparent worldwide lack of experience, it is very important that surgeons describe their experience on SILT and both short-term and long-term results of SILT are described and evaluated.

### Other techniques

Other intestinal lengthening techniques have been described, such as the double-barrel enteroplasty [19], the Kimura procedure [20], and distraction enterogenesis [21,22]. However, no papers have been published about these procedures in the last few years, the procedures are not eligible for general intestinal lengthening use, or these techniques have only been performed in an experimental setting.

## DISCUSSION

The goal of this overview paper is to describe the three main techniques of bowel lengthening procedures and show the results that have been described in the last few years. The amount of papers published about these techniques is unfortunately limited. Part of the scarcity in literature is because of the relatively rare incidence of the need for these procedures.

Autologous gut reconstruction (AGR) procedures are essential steps in gut failure care as it offers a high chance of improved enteral autonomy including less PN dependency and even PN independency [23]. Although it is cost-effective, these lengthening techniques are useful for selected patients, not all gut failure patients are candidates. In some cases, it is evident that a patient can never wean from PN due to the very short length of the bowel. These patients should be monitored closely while on PN. When line infections and thrombosis or liver failure become evident, patients should be referred early to a transplant center to evaluate them for transplantation. Prognosis after bowel transplantation becomes worse when performed in patients with poor condition and/or liver failure. So, although gut transplantation is a last resort, it should not be considered too late.

Therefore, a surgical algorithm for the management of patients with gut failure has been developed [23]. This algorithm can be used for the selection of patients for AGR or transplantation in relation to the organ failure(s) of the patient. For example, patients with nonreconstructable bowel might be eligible for bowel transplantation, but patients with an additional liver failure should be considered for liver and bowel transplantation. It should be mentioned that bowel transplantation still has major disadvantages, including high risks of complications and higher costs compared to AGR. Although AGR achieves better long-term survival than transplantation, transplantation shows better re-establishment of nutritional autonomy [23].

The choice for one of the described procedures is dependent on several surgeon-related as well as patient-related factors. First of all, the surgical experience must play an absolute major role in decision making, as these techniques are not easily performed and have small margins for disaster. The SILT procedure is probably the most technically challenging and should only be performed in centers with experience in these procedures or, in case of no clinical experience, it should be prepared in an animal model or on human cadavers before initiating such a program [17]. The STEP procedure is relatively easy to perform, and also has an advantage compared to LILT and SILT that the lumen of the bowel is not opened, reducing the amount of bacterial contamination and risk of intra-abdominal infection and subsequent abscess formation. A modified SILT where the mucosa is not cut has been proposed in an animal model that may circumvent abdominal contamination [24]. STEP can also be performed on the duodenum and has recently been described to be translated to the colon, serial transverse coloplasty or STCP [23]. Another variation on the STEP has been introduced, the modified STEP (MSTEP), where the mesenteric handling is avoided. This adjusted method has similar results to STEP, with 46% of patients achieving enteral autonomy and 19% surgical-related complications [25]. It has fewer contraindications, unlike the LILT procedure, which is for example not feasible in a very adhesive abdomen. LILT and SILT have the theoretical advantage of maintaining the muscle fiber orientation. Another advantage, at least in theory, of SILT, is that the surgeon can adjust the angle of the spiral cut in the bowel depending on the dilatation, in order to regulate the gained amount of length of bowel.

It should be questioned whether lengthening is always preferable and weighs up to the possible complications. In a recent comparing evaluation of the STEP and LILT, both procedures showed that almost half of the patients weaned off PN but have a mortality rate of 7% and 26% respectively [7]. So, indication and careful selection are very important in these patients.

## CONCLUSION

In conclusion, in patients with (ultra) short bowel syndrome and PN dependence an intestinal lengthening procedure should be considered. The main reason for considering these procedures should be to get nutritional autonomy or to reduce PN dependence and thereby reducing the chance of liver failure. Bacterial overgrowth and small bowel dilatation are probably the best surgical indications. Taking into account the complexity of these procedures and surgical complications in these already compromised patients, only centers with extensive experience in intestinal failure and complex abdominal surgery should perform these procedures. LILT, SILT, and STEP are the most frequently described lengthening procedures. Only few centers in the world have experience with more than one of these procedures. On the basis of the most recent literature, we conclude that STEP is currently the most performed procedure, probably because it is less complex to perform, the intestinal lumen is not opened and there is less chance of leakage and necrosis by damaging the mesentery. Therefore, we conclude that for most centers STEP should probably be the first-line procedure to perform. Although an international STEP data registry exists (https://apps.childrenshospital.org/externalClinical/STEP/) and a report was published in 2013 [26], recent reports of this and other procedures are scarce. For this reason, we also like to stimulate centers to describe their (retrospective) series with their results on intestinal lengthening procedures and report on the increase in length, decrease in PN dependence, complications and long-term outcome.

## Acknowledgements

*None.*

### Financial support and sponsorship

*None.*

### Conflicts of interest

*There are no conflicts of interest.*

## REFERENCES AND RECOMMENDED READING

Papers of particular interest, published within the annual period of review, have been highlighted as:

▪ of special interest
▪▪ of outstanding interest
